# A New Model for Ranking Schools of Public Health: The Public Health Academic Ranking

**DOI:** 10.3389/ijph.2024.1606684

**Published:** 2024-03-11

**Authors:** Adeline Dugerdil, Awa Babington-Ashaye, Murielle Bochud, Margaret Chan, Arnaud Chiolero, Andreas Gerber-Grote, Nino Künzli, Gilles Paradis, Milo Alan Puhan, L. Suzanne Suggs, Klazine Van der Horst, Gérard Escher, Antoine Flahault

**Affiliations:** ^1^ Institut de Santé Globale, Faculté de Médecine, Université de Genève, Geneva, Geneva, Switzerland; ^2^ Center for Primary Care and Public Health, University Center of General Medicine and Public Health, Lausanne, Vaud, Switzerland; ^3^ Vanke School of Public Health, Tsinghua University, Beijing, China; ^4^ Population Health Laboratory, Fribourg, Fribourg, Switzerland; ^5^ Institute of Primary Healthcare, Bern, Bern, Switzerland; ^6^ Department of Epidemiology, Biostatistics and Occupational Health, School of Population and Global Health, Faculty of Medicine and Health Sciences, McGill University, Montreal, QC, Canada; ^7^ School of Health Sciences, Zurich University of Applied Sciences, Winterthur, Zürich, Switzerland; ^8^ Swiss Tropical and Public Health Institute (Swiss TPH), Basel, Basel-Landschaft, Switzerland; ^9^ University of Basel, Basel, Switzerland; ^10^ Swiss School of Public Health (SSPH+) Directorate, Zürich, Zürich, Switzerland; ^11^ Department of Epidemiology, Institute of Epidemiology, Biostatistics and Prevention, Faculty of Medicine, University of Zurich, Zurich, Zurich, Switzerland; ^12^ Institute of Public Health and Institute of Communication and Public Policy, Lugano, Ticino, Switzerland; ^13^ Department of Health Professions, Bern University of Applied Sciences, Bern, Bern, Switzerland; ^14^ Geneva Science and Diplomacy Anticipator, Geneva, Geneva, Switzerland

**Keywords:** ranking methodology, university rankings, public health, schools of public health, public health academia, normalized bibliometric indicators

## Abstract

**Objectives:** As there is no ranking designed for schools of Public Health, the aim of this project was to create one.

**Methods:** To design the Public Health Academic Ranking (PHAR), we used the InCites Benchmarking and Analytics™ software and the Web Of Science™ Core Collection database. We collected bibliometric data on 26 schools of Public Health from each continent, between August and September 2022. We included 11 research indicators/scores, covering four criteria (productivity, quality, accessibility for readers, international collaboration), for the period 2017–2021. For the Swiss School of Public Health (SSPH+), a network gathering faculties across different universities, a specific methodology was used, with member-specific research queries.

**Results:** The five top schools of the PHAR were: London School of Hygiene and Tropical Medicine, Public Health Foundation of India, Harvard T.H. Chan School of Public Health, SSPH+, Johns Hopkins Bloomberg School of Public Health.

**Conclusion:** The PHAR allows worldwide bibliometric ordering of schools of Public Health. As this is a pilot project, the results must be taken with caution. This article aims to critically discuss its methodology and future improvements.

## Introduction

International university ranking systems were originally created to compare the performance of universities, but most have been criticized for not focusing enough on what is relevant to societies. Many rankings have been introduced since the creation of the Shanghai Ranking in 2003 [1], based on indicators from various fields such as research, education or teaching. They have several inherent well-described shortcomings [2, 3], and have often been interested in ranking universities as an entity, without adapting their indicators to the different disciplines, including Public Health [4]. Numerous shortcomings of current rankings have been highlighted in the literature, such as the choice, weighting and lack of inclusion of certain indicators, the issue of reputation surveys, the lack of inclusion of minorities and low-income countries, the bias in favor of English-based universities and the lack of transparency in methodology [3, 5, 6]. As stated by Vernon et al. (2018), who conducted a systematic review on ranking systems: “There is a need for a credible quality improvement movement in research that develops new measures, and is useful for institutions to evaluate and improve performance and societal value” [7].

According to the CDC Foundation, “Public health professionals try to prevent problems from happening or recurring through implementing educational programs, recommending policies, administering services and conducting research (…)” [8]. Although this definition is not exhaustive and Public Health includes several other areas (such as surveillance and outbreak investigation), it highlights the broad aspects covered by Public Health, the specificity of such a discipline, the societal impact it aims to achieve, as well as the particularity of research in this field (including community-based and observational studies). Furthermore, in the wake of the SARS-CoV-2 pandemic, it is widely recognized that more robust public health efforts are needed [9]. Strong Public Health requires not only excellent educational programs, but also a state-of-the-art research. As stated by Odone et al. (2017): “gaps exist between current public health needs and the extent to which public health workers are trained” [10]. In this context, a valid ranking is required to help all stakeholders improving this discipline, as a majority of university leaders use rankings to monitor the performance of their own institutions and to highlight their strengths in order to attract researchers and students from around the world [11].

Therefore, given the importance of Public Health, the common use of rankings in the academic world, and the demand for a valid ranking system, we designed and pilot tested a ranking for schools of Public Health. As an initial step, we did not seek to evaluate the fields of education and teaching, but developed a ranking based purely on bibliometric indicators evaluating the research field of Public Health. These indicators are only one means of evaluating a university and it is important to emphasize that the overall evaluation of a school must include many other parameters, which will be developed in later phases of this project.

## Methods

### Sources and Database

Although this definition cannot claim to be exhaustive regarding the broad scope of Public Health, “core disciplines include epidemiology, environmental health sciences, health policy and management, biostatistics, and social and behavioral aspects of health” [12]. In this context, we used the InCites Benchmarking and Analytics™ software (from Clarivate™ - named “software” in this paper) to collect data on schools of Public Health. The software is based on the publications metadata of the Web Of Science™ Core Collection database (herein named “database”) [13], that covers the main disciplines of Public Health mentioned above and many others [14]. This software only lists a small number of schools of Public Health. Other schools not listed in the software were selected from the database.

### Temporal Criteria for Indicators and Study Period

In order to obtain a meaningful ranking that is not subject to year-to-year variation, we used data from 2017 to 2021. We decided to select a 5 years time period as some of our criteria (particularly those concerned with citation numbers) require time to become relevant. The raw data needed for the construction of the ranking were collected between 1 August and 30 September 2022.

### Indicators and Scores Constituting the Ranking

This ranking is based on 11 bibliometric indicators and scores, from four different criteria (productivity, quality, accessibility for readers, international collaboration), which have been chosen because they are relevant for the discipline of Public Health. The term “indicators” refers to the raw data that have been collected, whereas the term “scores” refers to the normalized values of the raw data. While it seems obvious to include “productivity” and “quality” indicators/scores, Public Health being of high policy relevance, it also seemed essential to include indicators/scores related to the “accessibility for readers.” Finally, in areas such as pandemic management or migration policy for instance, Public Health is a discipline with a strong international focus, which is why the “international collaboration” indicator was significant. The indicators are described in Table 1.

**TABLE 1:** List of the 11 indicators used in the ranking, distributed in four criteria (Geneva, Switzerland, 2022).

No	Name of the indicator	Definition of the indicator
Productivity indicator		
1	Web of science documents	Number of documents in the web of science database, per school
Quality indicators		
2	Times cited	Number of times the set of publication has been cited
3	Average times cited	Average of citations per school, based on the number of web of science documents (=Times cited/Web of science documents)
4	Highly cited papers	Publications ranking in the top 1%, by citations for field and year
5	% highly cited papers	Percentage of publications in top 1%, by citations for field and year (=Highly cited papers/Web of science documents)
6	Hot papers	Publications ranking in top 0.1%, by citations for field and age
7	% hot papers	Percentage of publications in top 0.1%, by citations for field and age (=Hot papers/Web of science documents)
Accessibility for readers indicators		
8	All open access documents	Number of publications identified as open access (of any type)
9	% all open access documents	Percentage of publications identified as open access (=All open access documents/Web of science documents)
International collaboration indicators		
10	International collaboration	Publication containing one or more international co-authors
11	% international collaboration	Percentage of publication containing one or more international co-authors (=International collaboration/Web of science documents)

### Formulas Used in the Ranking

The formulas used for the ranking consisted of normalizing the indicators according to a “base 100” process (i.e., attribution (or assessment) of a score 100 to the school with the highest value of the indicator and assessment of the percentage of this score to the other schools, depending on the indicator values they had). The overall scores were then computed by summing up scores for the indicators in two different ways. The first formula consists of summing up all scores without any weighting. The second formula consists of the sum of six scores, with a weighting giving the same weight to each criterion. The formulas are detailed in Table 2.

**TABLE 2:** Formulas used for the ranking, based on indicators normalized in “base 100” (i.e., “score”). The score number corresponds to the number attributed to each indicator in Table 1 (Geneva, Switzerland, 2022).

Overall score N°	Formula (using indicators in “base 100,” i.e., “score”)
Overall score 1	Formula 1 = score 1 + score 2 + score 3 + score 4 + score 5 + score 6 + score 7 + score 8 + score 9 + score 10 + score 11
Overall score 2	Formula 2 = score 1 × 1 + score 3 x (1/3) + score 5 x(1/3) + score 7 x (1/3) + score 9 × 1 + score 11 × 1

The weighting given to each criterion of the two formulas is presented in Table 3. Formula 1 gives a significant weight to the “quality” criterion, while Formula 2 gives an equal weighting to all four criteria. Since all four criteria are of equal importance in Public Health, we selected Formula 2 to create the PHAR.

**TABLE 3:** Criterion weighting (in %) for Formula 1 and Formula 2 (Geneva, Switzerland, 2022).

Criterion	Criterion weighting in Formula 1 (%)	Criterion weighting in Formula 2 (%)	Difference in criterion weighting between formulas 1 and 2 (%)
Productivity	9	25	+16
Quality	55	25	−30
Accessibility for readers	18	25	+7
International collaboration	18	25	+7
Total	100	100	0

The raw data and the formulas for the ranking were downloaded and executed in M.S. Excel (version 2016).

### Study Population: Schools of Public Health Included in the Ranking

We collected data from 26 schools of Public Health worldwide. In this pilot project, our sub-sample of selected schools was based on the following criteria:

All the 12 schools of Public Health that were listed with specific dedicated affiliation in the software were selected. This search was conducted on the basis of keywords (in English and French) related to the discipline of Public Health. These keywords were: “Public Health,” “Santé Publique,” “Global Health,” “Tropical Medicine” (these last two keywords gave no results when translated in French). We added the Swiss School of Public Health (SSPH+), with the specific approach described below. We expanded the scope of the ranking, by adding 13 schools of Public Health from different geographic locations, not listed in the software, with the aim of including at least one school from each continent. It should be noted that none of the schools initially included in our sample were subsequently excluded from the study.

Since this is a pilot project, the choice of schools was necessarily limited, but will be completed in the future stages of the project. The list of schools included in the ranking is detailed in Table 4.

**TABLE 4:** List of the 26 schools of Public Health included in the ranking (according to countries, classified in alphabetical order) (Geneva, Switzerland, 2022).

N°	School name
Australia	
1	School of Public Health and Preventive Medicine, Monash University
Brazil	
2	School of Public Health, Universidade de Sao Paulo
Canada	
3	School of Population and Global Health, McGill University
4	Dalla Lana School of Public Health, University of Toronto
Chile	
5	School of Public Health, Universidade de Chile
Denmark	
6	School of Global Health, University of Copenhagen
France	
7	Ecole des Hautes Etudes en Sante Publique (EHESP)
India	
8	Public Health Foundation of India (PHFI)
Japan	
9	School of Public Health, Kyoto University
Senegal	
10	Institut de Santé et Développement, University Cheikh Anta Diop Dakar
Singapore	
11	Saw Swee Hock School of Public Health, National University of Singapore
South Africa	
12	School of Public Health, University of Cape Town
Spain	
13	Barcelona Institute for Global Health, ISGlobal
Switzerland	
14	Swiss School of Public Health (SSPH+)
Thailand	
15	Mahidol Oxford Tropical Medicine Research Unit (MORU), Tropical Health Network
United Kingdom	
16	London School of Hygiene and Tropical Medicine
17	Institute of Population Health, Liverpool School of Tropical Medicine
United States of America	
18	Harvard T.H. Chan School of Public Health, Harvard University
19	Johns Hopkins Bloomberg School of Public Health
20	Rollins School Public Health, Emory University
21	Colorado School of Public Health
22	University of Texas School of Public Health
23	Columbia University’s Mailman School of Public Health
24	UCLA Fielding School of Public Health
25	UNC Gillings School of Global Public Health
Vietnam	
26	Hanoi University of Public Health

### Research Queries Used in the Web of Science™ Core Collection Database

For the 14 schools that were not registered in the software, data were collected from the database. To find publications belonging to a certain school in the database, we developed a research query for each school. As an example, we take the Mailman School of Public Health, which is part of the Columbia University. In the software, this school is not registered, but Columbia University is listed (which includes the Mailman School of Public Health but also other departments). Therefore, we used the following query in the database, based on the affiliation (“OG”) and address (“AD”) of the Mailman School of Public Health:

OG=Columbia University AND AD=mailman OR AD=publ hlthAND 2021 or 2020 or 2019 or 2018 or 2017 Publication Years

The use of this method allowed the collection of data for all indicators, apart from the indicators 10 and 11. For those two indicators, we did an estimation, by using the “% International Collaboration” indicator that is attributed to the affiliated University listed in the software (we used the “% International Collaboration” attributed to Columbia University and applied it to the Mailman School of Public Health). All research queries are available in the Supplementary Appendix S1.

The SSPH+ is a unique case as it is, to our knowledge, the only “virtual campus” worldwide. Some of the other ranked schools gather different universities, but have a formal affiliation, such as the Colorado School of Public Health, so the situation is different. Given that none of the Swiss universities run schools of Public Health, the SSPH+ was created in 2005, building a virtual national faculty including all public health oriented scientists who belong to any faculty of one of the 12 universities of the SSPH+ (at the time of the ranking’s creation, SSPH+ included 12 universities). Given that the SSPH+ does not employ its virtual faculty members, most scientists do not use the SSPH+ as an affiliation. Thus, in contrast to all other schools of Public Health, the output of the SSPH+ faculty cannot be identified with the term “SSPH+“ Instead, the record of SSPH+ corresponds to the scientific output of the SSPH+ faculty members and their research groups. Thus, a different methodology was needed to derive the ranking of the SSPH+, explained by the use of the database and the research queries. We also asked several representatives of included universities to check the research queries used in the database, to avoid errors inherent to the research methodology. Furthermore, in order to avoid mistakenly counting duplicates in the data collected in the database (although this should rarely be the case having used the conjunction word “OR” to link the different queries for the universities within the SSPH+), we manually sampled the data (i.e., we selected the ten first articles published in the database from each of the 12 universities of the SSPH+) and checked the percentage of duplicates between publications from each SSPH+ university. As this percentage turns out to be minimal (1.67%), we did not make any correction to the collected data. Finally, we conducted a preliminary validation for one university using a subset of bibliometric outputs selected by the formula.

## Results

The results of the PHAR are detailed in Table 5. The range of scores awarded to the included schools extends from 128 (26th-ranked school) to 288 (1st-ranked school). For comparison purposes, we use the “score/unit” to compare scores between Formulas 1 and 2. The ranking according to Formula 1, the changes of rank between Formulas 1 and 2, and the detailed scores of each school are available in the Supplementary Appendices S2–S4.

**TABLE 5:** The Public Health Academic Ranking (i.e., according to Formula 2). To obtain the score/unit, Formula 2 score was divided by 4 (Formula 2 includes 6 scores with a respective weighting of 1, 3 × 1/3, 1 and 1) (Geneva, Switzerland, 2022).

Rank	School name/Country	Formula 2 score	Score/unit
1	London School of Hygiene and Tropical Medicine/United Kingdom	288	72.0
2	Public Health Foundation of India (PHFI)/India	276	68.9
3	Harvard T.H. Chan School of Public Health, Harvard University/United States	274	68.5
4	Swiss School of Public Health (SSPH+)/Switzerland	245	61.3
5	Johns Hopkins Bloomberg School of Public Health/United States	239	59.9
6	Mahidol Oxford Tropical Medicine Research Unit (MORU), Tropical Health Network/Thailand	236	59.1
7	School of Public Health, University of Cape Town/South Africa	233	58.2
8	Institute of Population Health, Liverpool School of Tropical Medicine/United Kingdom	229	57.3
9	Saw Swee Hock School of Public Health, National University of Singapore/Singapore	225	56.1
10	Barcelona Institute for Global Health, ISGlobal/Spain	224	56.0
11	School of Global Health, University of Copenhagen/Denmark	220	55.1
12	School of Population and Global Health, McGill University/Canada	217	54.4
13	Columbia University’s Mailman School of Public Health/United States	212	53.1
14	School of Public Health and Preventive Medicine, Monash University/Australia	201	50.3
15	UNC Gillings School of Global Public Health/United States	198	49.5
16	UCLA Fielding School of Public Health/United States	195	48.8
17	School of Public Health, Universidade de Sao Paulo/Brazil	194	48.4
18	School of Public Health, Universidade de Chile/Chile	184	46.0
19	Dalla Lana School of Public Health, University of Toronto/Canada	181	45.1
20	Hanoi University of Public Health/Vietnam	178	44.6
21	School of Public Health, Kyoto University/Japan	171	42.7
22	Rollins School of Public Health, Emory University/United States	170	42.4
23	Institut de Santé et Développement, University Cheikh Anta Diop Dakar/Senegal	168	42.1
24	University of Texas School of Public Health/United States	140	34.9
25	Ecole des Hautes Etudes en Sante Publique (EHESP)/France	136	34.0
26	Colorado School of Public Health/United States	128	31.9

The top five schools of the PHAR are: London School of Hygiene and Tropical Medicine (LSHTM), Public Health Foundation of India (PHFI), Harvard T.H. Chan School of Public Health, SSPH+, Johns Hopkins Bloomberg School of Public Health. The top five ranks are occupied by the same schools using Formulas 1 or 2, highlighting a stability between the two rankings even if the rank within the top five places changes.

The number of ranks that each school loses or gains by moving from Formula 1 to 2 is detailed in the Supplementary Appendix S3. The extremes of the ranking are the least subject to change (top five and bottom five ranks), while ranks six to 22 are the most subject to instability. The school with the greatest change in rank is the Mahidol Oxford Tropical Medicine Research Unit (MORU, +12 ranks), while six schools show only one change of ranks (positive or negative). The change of rank for the MORU can be understood as follows: it scores very well in the criteria “accessibility for readers” and “international collaboration” (weighting improving respectively of 7% between Formulas 1 and 2) whereas it scores less well for the “quality” criterion (weighting decreasing of 30% between Formulas 1 and 2).

## Discussion

In January 2023, Harvard Medical School withdrew from the U.S. News and World Report Ranking despite being ranked at the top of it, arguing that “Rankings cannot meaningfully reflect the high aspirations for educational excellence (…)” [15]. This movement of withdrawal highlights the shortcomings of existing rankings, as well as the need to review their methodology to create valid and useful rankings, to meet the demand of many stakeholders who use them as a decision-making tool [16, 17]. In addition, several initiatives have emerged, including the Declaration on Research Assessment (DORA) a decade ago, and recently the Coalition for Advancing Research Assessment (COARA), highlighting the need for change in research assessment and the move towards giving tribute to the impact of university-based research [18, 19]. As it is well known that universities rely in part on rankings to obtain funding, it seems clear that rankings can potentially have considerable implications for public health policies. A well-ranked school will find it easier to obtain funding, which in turn will generate a greater potential pool of Public Health experts at national level, and thus a definite impact on national public health policies. It seems therefore illusory to simply withdraw from a ranking; on the contrary, we need to collaborate and develop a valid, transparent and robust ranking that will enable us to promote this discipline, fundamentally important for our societies.

To our knowledge, the PHAR is the first international bibliometric university ranking system designed for academic Public Health. This pilot project is based on 11 bibliometric indicators and scores and ranks 26 schools of Public Health worldwide. Two different formulas were used for this pilot project, but only Formula 2 was finally selected for the PHAR, because of the same weight that this formula attributes to each criterion.

There are two main highlights in this ranking. First, although two of the top five schools were from the United States, the top ten schools included institutions from four continents. The presence of schools from India, Thailand, South Africa and Singapore is notable and may reflect the increasing importance of Public Health research in non-high income countries. The criteria of international collaboration may have allowed the identification of these schools as international leaders. Second, we can highlight two unexpected appearances in the top five ranks: the SSPH+ and the PHFI. The special case of the SSPH+ is detailed below. Concerning the PHFI, although this institute also gathers several schools, as it benefits from an affiliation listed in the software (implying that the authors sign with this affiliation), it was decided not to apply the same methodology as for the SSPH+. In fact, the PHFI corresponds to the situation of the Colorado School of Public Health. In spite of this difference, we notice that the PHFI ranks high. It is interesting to note that this school ranks exceptionally well not because of the “productivity” criterion (as might have been felt in view of the network bringing together numerous schools in the same country) but thanks to the “quality” criterion, particularly in view of the scores 3, 5 and 7, which are all at the maximum. As far as the SSPH+ is concerned, its high ranking is explained by the number of publications (underlining the importance of considering this network of highly productive schools) and also by the number of “open access” publications. As already mentioned, the PHAR helps to highlight these types of particular schools, which are often underestimated by traditional rankings.

### Strengths

Compared to the 2021 Global Ranking of Academic Subjects for Public Health [20], the top five ranks of the PHAR also contain the top three schools of the previously mentioned ranking. However, the SSPH+ is not classified in the Global Ranking of Academic Subjects for Public Health. Indeed, the specific issue about the SSPH+ is that this virtual school is underestimated internationally, because it is not taken in account in the existing rankings. Despite inherent structural differences of such “virtual faculty,” SSPH+ acts in many regards like a “real” school of Public Health, which is demonstrated by the extensive scientific collaboration in research (e.g., the “Corona Immunitas” study including more than 40 national studies on the immunity against SARS-CoV-2 in Switzerland [21]), the actions to improve Public Health education (e.g., the Inter-university Graduate Campus for PhD students [22]), or the strong professional network developed between these universities [23–25]. Thus, the SSPH+ provides a national network unifying this multidisciplinary field across its current universities. Therefore, one of the main strength of the PHAR is to offer visibility to this type of unique and decentralized school, which was previously unaccounted by the traditional rankings.

We perceive some strong arguments in favor of the PHAR.

First, the PHAR provides full transparency about the methodology used, allowing a reproducibility by any external stakeholders interested to replicate the ranking, as recommended by the Berlin Principles [26], and unlike some of the existing rankings whose methodology is sometimes described as a “black box approach” [27].

Second, the PHAR is an objective and robust ranking, based solely on bibliometric criteria. Although bibliometric indicators are subject to criticism, they have the advantage of being easily comparable and understandable, and to have a certain degree of objectivity [28–30].

Third, the PHAR is constructed on a well-recognized and reliable source of data [14]. Indeed, the software and database that were used in this ranking have a long experience in the field of scientometrics.

Finally, the PHAR aims to fill a gap in the ranking domain. As described by a previous work, to date, there does not exist any specific ranking designed for schools of Public Health or for “virtual schools” [4], therefore this project is innovative.

### Limitations

The PHAR, while innovative, does have its limitations.

First, this ranking is based purely on bibliometric criteria, focusing on the field of research. This choice was made in view of the importance of research in Public Health, as highlighted by McLaren et al. (2019) [31]. Nevertheless, we plan to include other indicators in later stages of the project (based for instance on the societal impact of schools), as proposed by Holmes (2021) regarding “third missions” indicators [6].

Second, the choice of the indicators can always be viewed as arbitrary, as highlighted regarding existing ranking shortcomings [2, 3]. Although these indicators already exist since longtime in the bibliometric field, we can still question their relevance: for instance, is publishing in Open Access journals a critical element of making papers “policy-relevant”? These questions require further research. Furthermore, the date of establishment of the ranked schools should always be taken into account, as the results of recently established schools may not be as pertinent as those of older schools.

A third limitation is that the queries used for the 14 schools for which data came from the database could include a potential source of error, and the definition of the term “school” may vary from a country to another, so these points could represent a bias. Nevertheless, we minimized this risk by asking some of the representatives of the ranked schools to do an external control of the research queries. In addition, although the use of these research queries may represent a source of error, we believe that this methodology is also one of the strengths of the ranking, as it allows the inclusion of schools usually overlooked by traditional rankings.

A fourth limitation of this pilot project is the need to restrict it to a manageable and rather small selection of schools of Public Health from all continents. Thus many schools are left out, including prestigious ones. Once algorithms of the PHAR are fully settled, the assessment may be easily extended. It is therefore important to emphasize that the accrediting bodies for schools of Public Health (e.g., APHEA in Europe), which contribute significantly to the evaluation (and accreditation) of certain schools, should not at present take the provisional ranking into account, as this could compromise the evaluation of the schools not included.

A fifth limitation of this ranking is that its scores do not take into account the size of the school. Thus, it is true that a large school will be better off in the number of publications by example than a smaller school. This certainly represents a future point of improvement.

Finally, the choice of the database may represent a bias, because Web of Science focuses mainly on publications written in English, and this represents a commonly criticized shortcoming regarding rankings [32]. Nevertheless, this database has the advantage of compiling numerous bibliometric indicators that allow for easy analysis of publications.

### Future Improvements

Future improvements include the integration of other evaluation domains (e.g., education, health economics), of others bibliometric indicators as well as non-bibliometric indicators, and of a non-English language database. Furthermore, this pilot project is limited in the number of included schools, but the aim is to progressively rank more schools worldwide and to classify schools according to the type of structure they present. As pointed out by de Leeuw (1995), there are at least eight different types of school of Public Health in Europe, and it would be interesting to be able to adapt the ranking to each of these types in order to bring greater refinement to the future ranking [33]. Some indicators specific to Public Health should also be developed, to make the ranking more specific to this discipline and to develop an evaluation of the impact of a school, possibly based on a participative and/or qualitative process. In conjunction with the previous step, a content analysis could also be designed to profile the different strengths of the schools.

Regarding the special case of the SSPH+, and although to our knowledge there is no official threshold for sensitivity and specificity in the bibliometric field, a sensitivity and specificity assessment was done on the articles selected by the research queries for the year 2021 of one member university. For the sensitivity analyses, five SSPH+ faculty members were chosen to compare their publication record, as listed on the institutional database, with the publications chosen by the algorithm. The queries had a sensitivity of 87% (13% of the publications actually made by those five SSPH+ affiliated members were missing). With a total of 33% articles included from authors of this university not formally affiliated with SSPH+, the specificity was 67%. In order to evaluate the impact of this specificity analysis, analyses for the SSPH+ ranking were recalculated by applying a “worst case” correction factor of 33% to the three indicators relating to publication volumes (indicators 1, 6, and 8). With this correction factor, the SSPH+’s rank has moved from the fourth to the sixth rank, highlighting that the SSPH+’s rank did not change significantly despite this correction (Supplementary Appendix S5). Moreover, algorithms with a higher sensitivity have the opposite effect on the ranking. Nevertheless, this point raises the question of a future methodological improvement regarding the research queries for SSPH+, which may need to be based on the names of the faculty members rather than the institutions and research fields alone.

Finally, an improvement could be made regarding the “international collaboration” indicator and the fact that 14 schools of the PHAR use an estimate of this indicator based on the university to which they are affiliated. Regarding this point, a second sensitivity analysis was conducted, excluding this indicator in order to analyze the change of ranks without including it. In this analysis, even if the ranks are changing for some schools, four of the top five ranks are still occupied by the same schools, highlighting a certain stability even when this indicator was excluded (Supplementary Appendix S6).

### Conclusion

The PHAR is the first international university bibliometric ranking designed for schools of Public Health, with a pilot project aiming to evaluate and focus on the research field of this discipline. As a result of initiatives such as COARA and DORA, research evaluation is being challenged and requires a change in the usual ways of thinking about this issue, integrating the impact of schools of Public Health on societies. However, and to provide an initial ranking of schools of Public Health, the main purpose of this article is to present and discuss the methodology used to create a valid ranking system, to underline its actual limitations, and to open the discussion for future improvements, as highlighted by Wilbers and Brankovic (2021) who stated “rankings are here to change and their status challenged” [34].
