# Supplementary material for: A New Model for Ranking Schools of Public Health: The Public Health Academic Ranking
Source: Int J Public Health. 2024 Mar 11;69:1606684. doi: 10.3389/ijph.2024.1606684 (PMC10961396; doi:10.3389/ijph.2024.1606684)
Supplement: Supplementary file 1 [file DataSheet1.pdf]

International Journal of Public Health

A new model for ranking schools of Public Health: The Public Health Academic Ranking – The PHAR

Supplementary Material

Appendix 1: Research queries used for the 14 schools of Public Health for which data was collected directly in the Web Of Science™ Core Collection database, between 1 August and 30 September 2022 (Geneva, Switzerland, 2022).

1. School of Public Health and Preventive Medicine, Monash University

(OG=(Monash University)) AND AD=(publ health) AND 2021 or 2020 or 2019 or 2018 or 2017 (Publication Years)

2. School of Public Health, Universidade de Sao Paulo

(OG=(Universidade de Sao Paulo)) AND AD=(publ health) AND 2021 or 2020 or 2019 or 2018 or 2017 (Publication Years)

3. School of Population and Global Health, McGill University

OG=(McGill University) AND (AD=(Global Hlth) OR AD=(populat) OR AD=(public Hlth)) AND 2021 or 2020 or 2019 or 2018 or 2017 (Publication Years)

4. Dalla Lana School of Public Health, University of Toronto

OG=(University of Toronto) AND (AD=(public hlth) OR AD=(Dalla Lana)) AND 2021 or 2020 or 2019 or 2018 or 2017 (Publication Years)

5. School of Public Health, Universidade de Chile

(OG=(Universidad de Chile)) AND AD=(publ health) AND 2021 or 2020 or 2019 or 2018 or 2017 (Publication Years)

6. School of Global Health, University of Copenhagen

OG=(University of Copenhagen) AND (AD=(publ health) OR AD=(global health)) AND 2021 or 2020 or 2019 or 2018 or 2017 (Publication Years)

7. School of Public Health, Kyoto University

(OG=(Kyoto University)) AND AD=(publ health) AND 2021 or 2020 or 2019 or 2018 or 2017 (Publication Years)

8. Institut de Santé et Développement, University Cheikh Anta Diop Dakar

OG=(University Cheikh Anta Diop Dakar) AND (AD=(publ health) OR AD=(sante publ)) AND 2021 or 2020 or 2019 or 2018 or 2017 (Publication Years)

9. Saw Swee Hock School of Public Health, National University of Singapore

OG=(National University of Singapore) AND (AD=(publ health) OR AD=(saw swee)) AND 2021 or 2020 or 2019 or 2018 or 2017 (Publication Years)

10. School of Public Health, University of Cape Town

OG=(University of Cape Town) AND AD=(publ health) AND 2021 or 2020 or 2019 or 2018 or 2017 (Publication Years)

11. Columbia University's Mailman School of Public Health

OG=(Columbia University) AND (AD=(mailman) OR AD=(publ hlth)) AND 2021 or 2020 or 2019 or 2018 or 2017 (Publication Years)

12. UCLA Fielding School of Public Health

OG=(University of California Los Angeles) AND (AD=(fielding) OR AD=(publ health)) AND 2021 or 2020 or 2019 or 2018 or 2017 (Publication Years)

13. UNC Gillings School of Global Public Health

OG=(University of North Carolina Chapel Hill) AND (AD=(publ health) OR AD=(gillings)) AND 2021 or 2020 or 2019 or 2018 or 2017 (Publication Years)

14. Swiss School of Public Health (SSPH+)

The following 12 equations were combined with the linking word "OR" to create the final research equation for the SSPH+ (in order to simplify the reading, we present them here separately):

a) University of Basel

OG=(Swiss Tropical & Public Health Institute) OR (((OG=(University of Basel)) AND AD=(Evidence-based Insurance Medicine)) OR AD=(EbIM)) OR (OG=(University of Basel)) AND AD=(biostat) OR (OG=(University of Basel)) AND AD=(pharmaceut med) OR (OG=(University of Basel)) AND AD=(Nursing) OR (OG=(University of Basel)) AND AD=(Sport) AND 2021 or 2020 or 2019 or 2018 or 2017 (Publication Years)

b) University of Lausanne

(OG=(University of Lausanne)) OR OG=(Centre Hospitalier Universitaire Vaudois (CHUV)) AND (AD=(IUMSP) OR AD=(prevent med) OR AD=(unisante) OR AD=(publ hlth)) AND 2021 or 2020 or 2019 or 2018 or 2017 (Publication Years)

c) University of Geneva

(OG=(University of Geneva)) AND AD=(sante globale) OR (OG=(University of Geneva)) AND AD=(epidemiol) OR (OG=(University of Geneva)) AND AD=(global health) AND 2021 or 2020 or 2019 or 2018 or 2017 (Publication Years)

d) University of Zürich

OG=(University of Zurich) AND (AD=(EBPI) OR AD=(biostat) OR AD=(Prevent) OR AD=(Evolutionary Med)) AND 2021 or 2020 or 2019 or 2018 or 2017 (Publication Years)

e) Zürcher Hochschule für Angewandte Wissenschaften (ZHAW)

((OG=(Zurich University of Applied Sciences) OR AD=(zhaw)) AND (AD=(health) OR AD=(psychol) OR AD=(law))) AND 2021 or 2020 or 2019 or 2018 or 2017 (Publication Years)

f) University of Bern

(OG=(University of Bern) OR OG=(University Hospital of Bern)) AND (AD=(ISPM) OR AD=(prevent med)) AND 2021 or 2020 or 2019 or 2018 or 2017 (Publication Years)

g) Berner Fachhochschule (BFH)

(AD=(Berner Fachhochschule)) OR AD=(BFH) AND (AD=(gesundheit) OR AD=(health)) AND 2021 or 2020 or 2019 or 2018 or 2017 (Publication Years)

h) University of Neuchâtel

OG=(University of Neuchatel) AND (AD=(droit sante) OR AD=(hlth law)) AND 2021 or 2020 or 2019 or 2018 or 2017 (Publication Years)

i) Università della Svizzera italiana (USI)

OG=(Universita della Svizzera Italiana) AND (AD=(IDEP) OR AD=(inst econ) OR AD=(publ hlth)) AND 2021 or 2020 or 2019 or 2018 or 2017 (Publication Years)

j) University of Applied Sciences and Arts of Southern Switzerland (SUPSI)

AD=(SUPSI) AND (AD=(sanita) OR AD=(health)) AND 2021 or 2020 or 2019 or 2018 or 2017 (Publication Years)

k) University of Fribourg

OG=(University of Fribourg) AND (AD=(Populat Hlth) OR AD=(Community Health)) AND 2021 or 2020 or 2019 or 2018 or 2017 (Publication Years)

l) University of Lucerne

(OG=(University of Lucerne)) AND AD=(health) AND 2021 or 2020 or 2019 or 2018 or 2017 (Publication Years)

Note: No difference in results was found with the terms "publ health" or "public hlth", which is why these two terms were used alternatively in the research queries.

Appendix 2: The ranking according to Formula 1. To obtain the score/unit, the Formula 1 score was divided by 11 (the Formula 1 includes 11 scores with a weighting of 1 for each score) (Geneva, Switzerland, 2022).

Rank	School name / Country	Formula 1 score	Score /unit
1	Harvard T.H. Chan School of Public Health, Harvard University / USA	812	73.8
2	London School of Hygiene & Tropical Medicine / UK	771	70.1
3	Swiss School of Public Health (SSPH+) / Switzerland	626	56.9
4	Johns Hopkins Bloomberg School of Public Health / USA	617	56.1
5	Public Health Foundation of India (PHFI) / India	535	48.7
6	Columbia University's Mailman School of Public Health / USA	530	48.2
7	School of Global Health, University of Copenhagen / Denmark	499	45.4
8	School of Public Health and Preventive Medicine, Monash University / Australia	469	42.7
9	UNC Gillings School of Global Public Health / USA	467	42.5
10	Saw Swee Hock School of Public Health, National University of Singapore / Singapore	458	41.6
11	School of Public Health, University of Cape Town / South Africa	452	41.1
12	School of Public Health, Universidade de Sao Paulo / Brazil	423	38.5
13	UCLA Fielding School of Public Health / USA	421	38.3
14	School of Population and Global Health, McGill University / Canada	402	36.5
15	Barcelona Institute for Global Health, ISGlobal / Spain	374	34.0
16	Institute of Population Health, Liverpool School of Tropical Medicine / UK	363	33.0
17	Dalla Lana School of Public Health, University of Toronto / Canada	342	31.1
18	Mahidol Oxford Tropical Medicine Research Unit (MORU), Tropical Health Network / Thailand	333	30.2
19	School of Public Health, Kyoto University / Japan	326	29.6
20	Rollins School of Public Health, Emory University / USA	317	28.8
21	School of Public Health, Universidade de Chile / Chile	290	26.3
22	Hanoi University of Public Health / Vietnam	241	21.9
23	University of Texas School of Public Health / USA	201	18.2
24	Colorado School of Public Health / USA	198	18.0
25	Institut de Santé et Développement, University Cheikh Anta Diop Dakar / Senegal	184	16.7
26	Ecole des Hautes Etudes en Sante Publique (EHESP) / France	163	14.8

Appendix 3: Change of rank when passing from Formula 1 to Formula 2. E.g.: +2 means that the school has moved up 2 ranks from Formula 1 to Formula 2 (Geneva, Switzerland, 2022).

**School name**	**Change of rank: Formula 1 → Formula 2**
London School of Hygiene & Tropical Medicine	+1
Public Health Foundation of India (PHFI)	+3
Harvard T.H. Chan School of Public Health, Harvard University	-2
Swiss School of Public Health (SSPH+)	-1
Johns Hopkins Bloomberg School of Public Health	-1
Mahidol Oxford Tropical Medicine Research Unit (MORU), Tropical Health Network	+12
School of Public Health, University of Cape Town	+4
Institute of Population Health, Liverpool School of Tropical Medicine	+8
Saw Swee Hock School of Public Health, National University of Singapore	+1
Barcelona Institute for Global Health, ISGlobal	+5
School of Global Health, University of Copenhagen	-4
School of Population and Global Health, McGill University	+2
Columbia University’s Mailman School of Public Health	-7
School of Public Health and Preventive Medicine, Monash University	-6
UNC Gillings School of Global Public Health	-6
UCLA Fielding School of Public Health	-3
School of Public Health, Universidade de Sao Paulo	-5
School of Public Health, Universidade de Chile	+3
Dalla Lana School of Public Health, University of Toronto	-2
Hanoi University of Public Health	+2
School of Public Health, Kyoto University	-2
Rollins School of Public Health, Emory University	-2
Institut de Santé et Développement, University Cheikh Anta Diop Dakar	+2
University of Texas School of Public Health	-1
Ecole des Hautes Etudes en Sante Publique (EHESP)	+1
Colorado School of Public Health	-2

Appendix 4: Detailed scores according to Formula 1 and Formula 2 , for the 26 schools of Public Health included in the ranking. The term "indicators" refers to the raw data that have been collected, whereas the term "scores" refers to the normalized value of the raw data (i.e. "base 100") (Geneva, Switzerland, 2022).

	**Indicator/ score 1** *Web of Science Documents*	**Indicator/ score 2** *Times Cited*	**Indicator/ score 3** *Average Times Cited*	**Indicator/ score 4** *Highly Cited Papers*	**Indicator/ score 5** *% Highly Cited Papers*	**Indicator/ score 6** *Hot Papers*	**Indicator/ score 7** *% Hot Papers*	**Indicator/ score 8** *All Open Access Documents*	**Indicator/ score 9** *% All Open Access Documents*	**Indicator/ score 10** *International Collaboration*	**Indicator/ score 11** *%International Collaboration*	**Formula 1 score**	**Formula 2 score**
**Schools for which data was collected in the InCites Benchmarking and Analytics™ software**													
Harvard T.H. Chan School of Public Health	21 734	449 265	21	710	3	47	0	15 402	71	12 587	58		
Harvard T.H. Chan School of Public Health - **Base 100**	100	100	32	100	37	84	25	100	82	92	61	812	274
Johns Hopkins Bloomberg School of Public Health	19 449	301 626	16	417	2	31	0	13 486	69	8 836	45		
Johns Hopkins Bloomberg School of Public Health - **Base 100**	89	67	24	59	24	55	18	88	80	64	48	617	239
London School of Hygiene & Tropical Medicine	17 595	299 800	17	438	2	56	0	14 439	82	13 729	78		

	**Indicator/ score 1** *Web of Science Documents*	**Indicator/ score 2** *Times Cited*	**Indicator/ score 3** *Average Times Cited*	**Indicator/ score 4** *Highly Cited Papers*	**Indicator/ score 5** *% Highly Cited Papers*	**Indicator/ score 6** *Hot Papers*	**Indicator/ score 7** *% Hot Papers*	**Indicator/ score 8** *All Open Access Documents*	**Indicator/ score 9** *% All Open Access Documents*	**Indicator/ score 10** *International Collaboration*	**Indicator/ score 11** *%International Collaboration*	**Formula 1 score**	**Formula 2 score**
London School of Hygiene & Tropical Medicine - **Base 100**	81	67	27	62	28	100	36	94	95	100	82	771	288
Rollins School of Public Health	6 068	86 421	14	124	2	12	0	4 275	70	2 185	36		
Rollins School of Public Health - **Base 100**	28	19	22	17	23	21	22	28	82	16	38	317	170
Institute of Population Health, Liverpool School of Tropical Medicine	3 254	46 607	14	57	2	12	0	2 717	84	2 789	86		
Institute of Population Health, Liverpool School of Tropical Medicine - **Base 100**	15	10	22	8	20	21	42	18	97	20	90	363	229

	**Indicator/ score 1** *Web of Science Documents*	**Indicator/ score 2** *Times Cited*	**Indicator/ score 3** *Average Times Cited*	**Indicator/ score 4** *Highly Cited Papers*	**Indicator/ score 5** *% Highly Cited Papers*	**Indicator/ score 6** *Hot Papers*	**Indicator/ score 7** *% Hot Papers*	**Indicator/ score 8** *All Open Access Documents*	**Indicator/ score 9** *% All Open Access Documents*	**Indicator/ score 10** *International Collaboration*	**Indicator/ score 11** *%International Collaboration*	**Formula 1 score**	**Formula 2 score**
Colorado School of Public Health	3 202	41 241	13	44	1	3	0	2 054	64	718	22		
Colorado School of Public Health - **Base 100**	15	9	20	6	15	5	10	13	74	5	23	198	128
Barcelona Institute for Global Health, ISGlobal	3 016	79 395	26	97	3	8	0	2 260	75	2 514	83		
Barcelona Institute for Global Health, ISGlobal - **Base 100**	14	18	41	14	36	14	30	15	87	18	87	374	224
University of Texas School of Public Health	2 267	36 158	16	44	2	-	-	1 508	67	791	35		
University of Texas School of Public Health - **Base 100**	10	8	25	6	22	-	-	10	77	6	37	201	140
Mahidol Oxford Tropical Medicine Research Unit,	1 409	21 798	15	33	2	5	0	1 218	86	1 345	95		

	**Indicator/ score 1** *Web of Science Documents*	**Indicator/ score 2** *Times Cited*	**Indicator/ score 3** *Average Times Cited*	**Indicator/ score 4** *Highly Cited Papers*	**Indicator/ score 5** *% Highly Cited Papers*	**Indicator/ score 6** *Hot Papers*	**Indicator/ score 7** *% Hot Papers*	**Indicator/ score 8** *All Open Access Documents*	**Indicator/ score 9** *% All Open Access Documents*	**Indicator/ score 10** *International Collaboration*	**Indicator/ score 11** *%International Collaboration*	**Formula 1 score**	**Formula 2 score**
Tropical Health Network													
Mahidol Oxford Tropical Medicine Research Unit, Tropical Health Network – **Base 100**	6	5	24	5	26	9	39	8	100	10	100	333	236
Ecole des Hautes Etudes en Sante Publique	1 122	8 494	8	11	1	-	-	767	68	472	42		
Ecole des Hautes Etudes en Sante Publique - **Base 100**	5	2	12	2	11	-	-	5	79	3	44	163	136
Hanoi University of Public Health	461	15 455	34	11	2	-	-	308	67	318	69		
Hanoi University of Public Health - **Base 100**	2	3	53	2	27	-	-	2	77	2	72	241	178

	Indicator/ score 1<br>*Web of Science Documents*	Indicator/ score 2<br>*Times Cited*	Indicator/ score 3<br>*Average Times Cited*	Indicator/ score 4<br>*Highly Cited Papers*	Indicator/ score 5<br>*% Highly Cited Papers*	Indicator/ score 6<br>*Hot Papers*	Indicator/ score 7<br>*% Hot Papers*	Indicator/ score 8<br>*All Open Access Documents*	Indicator/ score 9<br>*% All Open Access Documents*	Indicator/ score 10<br>*International Collaboration*	Indicator/ score 11<br>*%International Collaboration*	Formula 1 score	Formula 2 score
Public Health Foundation of India	1 118	71 232	64	99	9	10	1	873	78	855	76		
Public Health Foundation of India - **Base 100**	5	16	100	14	100	18	100	6	90	6	80	535	276
**Schools for which data was collected from the Web Of Science™ Core Collection database**													
School of Public Health, University of Cape Town	3 380	104 314	31	147	4	19	1	2 785	82	2 206	65		
School of Public Health, University of Cape Town - **Base 100**	16	23	48	21	49	34	63	18	95	16	68	452	233
Saw Swee Hock School of Public Health, National University of Singapore	3 885	116 415	30	182	5	21	1	2 866	74	2 515	65		
Saw Swee Hock School of Public Health,	18	26	47	26	53	38	61	19	85	18	68	458	225

	**Indicator/ score 1** *Web of Science Documents*	**Indicator/ score 2** *Times Cited*	**Indicator/ score 3** *Average Times Cited*	**Indicator/ score 4** *Highly Cited Papers*	**Indicator/ score 5** *% Highly Cited Papers*	**Indicator/ score 6** *Hot Papers*	**Indicator/ score 7** *% Hot Papers*	**Indicator/ score 8** *All Open Access Documents*	**Indicator/ score 9** *% All Open Access Documents*	**Indicator/ score 10** *International Collaboration*	**Indicator/ score 11** *%International Collaboration*	**Formula 1 score**	**Formula 2 score**
National University of Singapore - **Base 100**													
School of Public Health and Preventive Medicine, Monash University	8 136	175 656	22	238	3	32	0	4 911	60	4 425	54		
School of Public Health and Preventive Medicine, Monash University - **Base 100**	37	39	34	34	33	57	44	32	70	32	57	469	201
School of Public Health, Kyoto University	1 963	67 473	34	88	4	8	0	1 273	65	689	35		
School of Public Health, Kyoto University - **Base 100**	9	15	54	12	51	14	46	8	75	5	37	326	171
School of Public Health, Universidade de Chile	624	19 115	31	27	4	2	0	424	68	347	56		

	Indicator/ score 1 *Web of Science Documents*	Indicator/ score 2 *Times Cited*	Indicator/ score 3 *Average Times Cited*	Indicator/ score 4 *Highly Cited Papers*	Indicator/ score 5 *% Highly Cited Papers*	Indicator/ score 6 *Hot Papers*	Indicator/ score 7 *% Hot Papers*	Indicator/ score 8 *All Open Access Documents*	Indicator/ score 9 *% All Open Access Documents*	Indicator/ score 10 *International Collaboration*	Indicator/ score 11 *%International Collaboration*	**Formula 1 score**	**Formula 2 score**
School of Public Health, Universidade de Chile - **Base 100**	3	4	48	4	49	4	36	3	79	3	58	290	184
School of Public Health, Universidade de Sao Paulo	3 026	112 179	37	184	6	17	1	1 874	62	1 296	43		
School of Public Health, Universidade de Sao Paulo - **Base 100**	14	25	58	26	69	30	63	12	72	9	45	423	194
Institut de Santé et Développement, University Cheikh Anta Diop Dakar	186	1 651	9	1	1	-	-	135	73	136	73		
Institut de Santé et Développement, University Cheikh Anta Diop Dakar - **Base 100**	1	0	14	0	6	-	-	1	84	1	77	184	168

	Indicator/ score 1<br>*Web of Science Documents*	Indicator/ score 2<br>*Times Cited*	Indicator/ score 3<br>*Average Times Cited*	Indicator/ score 4<br>*Highly Cited Papers*	Indicator/ score 5<br>*% Highly Cited Papers*	Indicator/ score 6<br>*Hot Papers*	Indicator/ score 7<br>*% Hot Papers*	Indicator/ score 8<br>*All Open Access Documents*	Indicator/ score 9<br>*% All Open Access Documents*	Indicator/ score 10<br>*International Collaboration*	Indicator/ score 11<br>*%International Collaboration*	Formula 1 score	Formula 2 score
Swiss School of Public health	13 425	269 436	20	406	3	36	0	9 538	71	10 168	66		
Swiss School of Public health - **Base 100**	62	60	31	57	34	64	30	62	82	74	69	626	245
Columbia University's Mailman School of Public Health	10 368	235 509	23	369	4	33	0	7 585	73	4 230	41		
Columbia University's Mailman School of Public Health - **Base 100**	48	52	36	52	40	59	36	49	85	31	43	530	212
UCLA Fielding School of Public Health	7 404	172 155	23	239	3	17	0	5 570	75	2 923	39		
UCLA Fielding School of Public Health - **Base 100**	34	38	36	34	36	30	26	36	87	21	41	421	195
UNC Gillings School of Global Public Health	11 834	237 057	20	303	3	21	0	8 589	73	3 710	31		

	**Indicator/ score 1** *Web of Science Documents*	**Indicator/ score 2** *Times Cited*	**Indicator/ score 3** *Average Times Cited*	**Indicator/ score 4** *Highly Cited Papers*	**Indicator/ score 5** *% Highly Cited Papers*	**Indicator/ score 6** *Hot Papers*	**Indicator/ score 7** *% Hot Papers*	**Indicator/ score 8** *All Open Access Documents*	**Indicator/ score 9** *% All Open Access Documents*	**Indicator/ score 10** *International Collaboration*	**Indicator/ score 11** *%International Collaboration*	**Formula 1 score**	**Formula 2 score**
UNC Gillings School of Global Public Health - **Base 100**	54	53	31	43	29	38	20	56	84	27	33	467	198
School of Population and Global Health, McGill University	1 886	85 269	45	123	7	6	0	1 469	78	1 054	56		
School of Population and Global Health, McGill University - **Base 100**	9	19	71	17	74	11	36	10	90	8	59	402	217
Dalla Lana School of Public Health, University of Toronto	6 230	108 831	17	130	2	12	0	3 869	62	3 323	53		
Dalla Lana School of Public Health, University of Toronto - **Base 100**	29	24	27	18	24	21	22	25	72	24	56	342	181

	Indicator/ score 1 *Web of Science Documents*	Indicator/ score 2 *Times Cited*	Indicator/ score 3 *Average Times Cited*	Indicator/ score 4 *Highly Cited Papers*	Indicator/ score 5 *% Highly Cited Papers*	Indicator/ score 6 *Hot Papers*	Indicator/ score 7 *% Hot Papers*	Indicator/ score 8 *All Open Access Documents*	Indicator/ score 9 *% All Open Access Documents*	Indicator/ score 10 *International Collaboration*	Indicator/ score 11 *%International Collaboration*	Formula 1 score	Formula 2 score
School of Global Health, University of Copenhagen	7 601	190 156	25	290	4	26	0	5 215	69	4 771	63		
School of Global Health, University of Copenhagen - **Base 100**	35	42	39	41	43	46	38	34	79	35	66	499	220

Appendix 5: The ranking (i.e. according to Formula 2), with an adaptation to the specificity calculation done on the Swiss School of Public Health plus (SSPH+). For this adaptation of the SSPH+ scores, the raw data for indicators and scores 1, 6 and 8 were reduced by a factor of 33% each (specificity of 67%, i.e. false positive of 33%). To obtain the score/unit, the Formula 2 score was divided by 4 (the Formula 2 includes 6 scores with a respective weighting of 1, 3 x 1/3, 1 and 1) (Geneva, Switzerland, 2022).

Rank	School name / Country	Formula 2 score	Score /unit
1	London School of Hygiene & Tropical Medicine / UK	288	72.0
2	Public Health Foundation of India (PHFI) / India	276	68.9
3	Harvard T.H. Chan School of Public Health, Harvard University / USA	274	68.5
4	Johns Hopkins Bloomberg School of Public Health / USA	239	59.9
5	Mahidol Oxford Tropical Medicine Research Unit (MORU), Tropical Health Network / Thailand	236	59.1
6	Swiss School of Public Health (SSPH+) / Switzerland	235	58.9
7	School of Public Health, University of Cape Town / South Africa	233	58.2
8	Institute of Population Health, Liverpool School of Tropical Medicine / UK	229	57.3
9	Saw Swee Hock School of Public Health, National University of Singapore / Singapore	225	56.1
10	Barcelona Institute for Global Health, ISGlobal / Spain	224	56.0
11	School of Global Health, University of Copenhagen / Denmark	220	55.1
12	School of Population and Global Health, McGill University / Canada	217	54.4
13	Columbia University's Mailman School of Public Health / USA	212	53.1
14	School of Public Health and Preventive Medicine, Monash University / Australia	201	50.3
15	UNC Gillings School of Global Public Health / USA	198	49.5
16	UCLA Fielding School of Public Health / USA	195	48.8
17	School of Public Health, Universidade de Sao Paulo / Brazil	194	48.4
18	School of Public Health, Universidade de Chile / Chile	184	46.0
19	Dalla Lana School of Public Health, University of Toronto / Canada	181	45.1
20	Hanoi University of Public Health / Vietnam	178	44.6
21	School of Public Health, Kyoto University / Japan	171	42.7
22	Rollins School of Public Health, Emory University / USA	170	42.4
23	Institut de Santé et Développement, University Cheikh Anta Diop Dakar / Senegal	168	42.1
24	University of Texas School of Public Health / USA	140	34.9
25	Ecole des Hautes Etudes en Sante Publique (EHESP) / France	136	34.0
26	Colorado School of Public Health / USA	128	31.9

Appendix 6: The ranking (i.e. according to an adapted version of Formula 2). For this ranking, the analyses are based on an adapted version of Formula 2 (without score 11, “international collaboration”), with also the Swiss School of Public Health plus (SSPH+) scores adapted for the calculation of specificity (see Appendix 5). To obtain the score/unit, the Formula 2 score was divided by 3 (the adapted Formula 2 includes 5 scores with a respective weighting of 1, 3 x 1/3, 1) (Geneva, Switzerland, 2022).

Rank	School name	Formula 2 score	Score /unit
1	Harvard T.H. Chan School of Public Health, Harvard University / USA	213	71.1
2	London School of Hygiene & Tropical Medicine / UK	206	68.7
3	Public Health Foundation of India (PHFI) / India	195	65.2
4	Johns Hopkins Bloomberg School of Public Health / USA	192	64.0
5	Columbia University’s Mailman School of Public Health / USA	170	56.5
6	Swiss School of Public Health (SSPH+) / Switzerland	166	55.4
7	UNC Gillings School of Global Public Health / USA	165	55.1
8	School of Public Health, University of Cape Town / South Africa	164	54.8
9	School of Population and Global Health, McGill University / Canada	159	53.0
10	Saw Swee Hock School of Public Health, National University of Singapore / Singapore	157	52.3
11	School of Global Health, University of Copenhagen / Denmark	155	51.5
12	UCLA Fielding School of Public Health / USA	154	51.3
13	School of Public Health, Universidade de Sao Paulo / Brazil	149	49.6
14	School of Public Health and Preventive Medicine, Monash University / Australia	144	48.1
15	Institute of Population Health, Liverpool School of Tropical Medicine / UK	140	46.5
16	Barcelona Institute for Global Health, ISGlobal / Spain	137	45.5
17	Mahidol Oxford Tropical Medicine Research Unit (MORU), Tropical Health Network / Thailand	136	45.5
18	School of Public Health, Kyoto University / Japan	134	44.7
19	Rollins School of Public Health, Emory University / USA	132	44.0
20	School of Public Health, Universidade de Chile / Chile	126	41.9
21	Dalla Lana School of Public Health, University of Toronto / Canada	125	41.6
22	Hanoi University of Public Health / Vietnam	106	35.3
23	Colorado School of Public Health / USA	104	34.7
24	University of Texas School of Public Health / USA	103	34.3
25	Ecole des Hautes Etudes en Sante Publique (EHESP) / France	92	30.6
26	Institut de Santé et Développement, University Cheikh Anta Diop Dakar / Senegal	91	30.5
